# Lymphovascular invasion represents a superior prognostic and predictive pathological factor of the duration of adjuvant chemotherapy for stage III colon cancer patients

**DOI:** 10.1186/s12885-022-10416-7

**Published:** 2023-01-03

**Authors:** Linjie Zhang, Yuxiang Deng, Songran Liu, Weili Zhang, Zhigang Hong, Zhenhai Lu, Zhizhong Pan, Xiaojun Wu, Jianhong Peng

**Affiliations:** 1 Department of Colorectal Surgery, Sun Yat-sen University Cancer Center; State Key Laboratory of Oncology in South China; Collaborative Innovation Center for Cancer Medicine, 651 Dongfeng Road East, Guangdong 510060 Guangzhou, P. R. China; 2 Department of Thyroid and Breast Surgery, Peking University Shenzhen Hospital, Shenzhen Peking University-The Hong Kong University of Science and Technology Medical Center, 518000 Shenzhen, P. R. China; 3 Department of Pathology, Sun Yat-sen University Cancer Center; State Key Laboratory of Oncology in South China; Collaborative Innovation Center for Cancer Medicine, 651 Dongfeng Road East, Guangdong 510060 Guangzhou, P. R. China

**Keywords:** Lymphovascular invasion, Perineural invasion, Adjuvant chemotherapy, Stage III colon cancer, Prognosis

## Abstract

**Background:**

Lymphovascular invasion (LVI) and perineural invasion (PNI) can indicate poor survival outcomes in colorectal cancer, but few studies have focused on stage III colon cancer. The current study aimed to confirm the prognostic value of LVI and PNI and identify patients who could benefit from a complete duration of adjuvant chemotherapy based on the two pathological factors.

**Methods:**

We enrolled 402 consecutive patients with stage III colon cancer who received colon tumor resection from November 2007 to June 2016 at Sun Yat-sen University Cancer Center. Survival analyses were performed by using Kaplan–Meier method with log-rank tests. Risk factors related to disease-free survival (DFS) and overall survival (OS) were identified through Cox proportional hazards analysis.

**Results:**

141 (35.1%) patients presented with LVI, and 108 (26.9%) patients with PNI. The LVI-positive group was associated with poorer 3-year DFS (86.5% vs. 76.3%, *P* = 0.001) and OS (96.0% vs. 89.1%, *P* = 0.003) rates compared with the LVI-negative group. The PNI-positive group showed a worse outcome compared with the PNI-negative group in 3-year DFS rate (72.5% vs. 86.7%, *P* < 0.001). Moreover, LVI-positive group present better 3-year DFS and OS rate in patients completing 6–8 cycles of adjuvant chemotherapy than those less than 6 cycles (3-year DFS: 80.0% vs. 64.9%, *P* = 0.019; 3-year OS: 93.2% vs. 76.3%, *P* = 0.002).

**Conclusions:**

LVI is a superior prognostic factor to PNI in stage III colon cancer patients undergoing curative treatment. PNI status can noly predict the 3-year DFS wihout affecting the 3-year OS. Furthermore, LVI also represents an effective indicator for adjuvant chemotherapy duration.

## Background

To date, the combination of curative surgery and oxaliplatin-based chemotherapy is well recommended as the classical treatment strategy for stage III colon cancer [1, 2]. A 6-month duration of adjuvant chemotherapy was previously recommended for all stage III colon cancer patients [3–6]. However, two major problems remain to be solved in clinical practice. 20 to 40% of patients fail to benefit from adjuvant chemotherapy and ultimately develop postoperative metastases [7, 8]. Moreover, chemotherapy-related toxicity, especially oxaliplatin-based sensory neurotoxicity, caused 50% of patients to be unable to finish the entire planned duration of adjuvant chemotherapy [9]. Recently, International Duration Evaluation of Adjuvant Therapy (IDEA) trial introduced risk definitions for stage III colon cancer to guide the duration of adjuvant chemotherapy. The final results indicated that 3-month XELOX adjuvant chemotherapy appeared to be sufficient for low-risk patients (T1-3 N1 disease) but not high-risk patients (T4, N2, or both diseases) [10]. Although the TNM stage is essential for colon cancer management, it seems insufficient to determine the specific risk patients with stage III colon cancer will benefit from adjuvant chemotherapy. The advancement of methods to better classify patients with stage III colon cancer may help develop more personalized strategies which allow more patients truly benefit from chemotherapy and avoid excessive toxic chemotherapy that is unlikely to give any survival benefits [11]. Herein, identifying important pathological factors is necessary to aid in risk stratification and select patients who need appropriate adjuvant treatment.

Current guidelines note that two tumor-specific parameters including lymphovascular invasion (LVI) and perineural invasion (PNI), should be reported in the pathological stage for colon cancer [3, 12]. LVI is recognized that tumor cells are involved in small endothelium-lined lymphatic or vascular channels, which is indicated as an early and obligatory step of tumor metastasis [13, 14]. PNI is a pathologic process of tumor growth within the nerves and nerve sheaths and is a pathological marker for a more aggressive tumor phenotype [15, 16]. Accumulating evidence has well demonstrated that either or both pathological factors indicate a poor survival outcome in colorectal cancer (CRC) [17, 18]. Currently, most studies have focused on investigating the prognostic role of the two pathological factors in stage I and stage II CRC and identified both as indications for adjuvant chemotherapy [19, 20]. However, only a limited number of studies reported the prognostic value of LVI and PNI in stage III colon cancer [21, 22]. The actual prognostic effect of LVI and PNI remains unclear in stage III colon cancer patients undergoing curative treatment. In addition, evidence on whether LVI and PNI could serve as markers for the duration of adjuvant chemotherapy is still lacking.

To address these two issues, the current study aimed to demonstrate the prognostic effect of LVI and PNI in stage III colon cancer patients receiving curative surgery followed by adjuvant chemotherapy. Subsequently, we aimed to identify the specific group of patients who could benefit from a total duration of adjuvant chemotherapy according to the presence of LVI and PNI.

## Materials and methods

### Patients

A total of 402 consecutive patients with stage III colon cancer who underwent primary tumor resection between November 2007 and June 2016 at Sun Yat-sen University Cancer Center were included in this retrospective study. The patients were enrolled according to the following criteria: (1) pathologically diagnosed as colon adenocarcinoma; (2) colon tumor curative resection; (3) adjuvant chemotherapy with the XELOX regimen (oxaliplatin plus capecitabine); (4) complete pathological data with definite LVI and PNI statuses; (5) no preoperative anticancer treatment; (6) American Society of Anesthesiologists class I–II; and (7) postoperative follow-up at least 3 months after delivery of the first cycle of chemotherapy. The clinical information, including demographics, tumor characteristics, treatment details, and follow-up data, were carefully collected from the electronic medical record system. Right-sided colon cancer was defined as the tumor located in cecum, ascending, hepatic flexure, and transverse colon, whereas left-sided colon cancer was recognized as the tumor in splenic flexure, descending, and sigmoid colon. The current study was conducted based on the ethical standards of the World Medical Association Declaration of Helsinki. This study was approved by the Institutional Research Ethics Committee of Sun Yat-sen University Cancer Center (approval number: B2022-790-01). All patient data were documented confidentially.

### Treatments

All the patients underwent curative resection of the colon tumor by performing standard complete mesocolic excision and regional lymphadenectomy. The initial adjuvant chemotherapy was performed 3–8 weeks for all the patients after colon tumor resection. The XELOX regimen was administered as 3-week cycle chemotherapy as 130 mg/m^2^ oxaliplatin on day 1 combined with 1000 mg/m^2^ capecitabine twice daily on days 1–14 at an interval of 7 days. The continued administration of the XELOX regimen of adjuvant chemotherapy depended on the patient’s general status, the toxicity of the chemotherapy, or the patient’s tolerance to the subsequent cycle of chemotherapy.

### Pathologic analysis

**Fig. 1:**
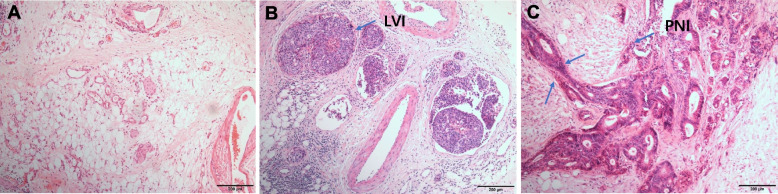
**A** The normal nerve bundles and vessels; **B** The presence of lymphovascular invasion; **C** The presence of perineural invasion. (blue arrow)

Each tumor resection specimen was reviewed by two independent pathologists (Songran Liu and Shixun Lu). All cases were pathologically staged referring to the 8th edition of the American Joint Committee on Cancer (AJCC) staging system. Hematoxylin and eosin staining was used to evaluate the LVI status without other special stains. LVI was diagnosed as the presence of tumor cells within the small endothelium-lined lymphatic or vascular channels [14]. PNI was diagnosed as tumor invasion in, around, and through nerves and nerve sheaths (Fig. 1) [15]. In addition, the statuses of proximal and distal margins, lymph node metastasis, and tumor differentiation were assessed in line with current guidelines [3, 12].

### Postoperative follow-up

The follow-up was conducted every 3 months for the first 2 years and then semiannually for the subsequent 3 years after surgery through clinical visits. The clinical visit items included abdomen examinations, detection of serum carcinoembryonic antigen (CEA) and carbohydrate antigen 19-9 (CA19-9), chest/abdominal/pelvic CT, and colonoscopy. Disease-free survival (DFS) was the interval from tumor resection to the date of disease recurrence, death, or the last follow-up. Overall survival (OS) was the interval from tumor resection to the date of death from any cause or the last follow-up. The final follow-up visit conducted in July 2019.

### Statistical analysis

Statistical analyses were conducted through SPSS 20.0 software (IBM, Chicago, IL, USA) and GraphPad Prism 7 software (GraphPad Software, Inc., San Diego, CA, USA). Continuous variables are presented as the median (range), while categorical variables are presented as percentages, which were compared by using the chi-square (χ2) test. The Kaplan–Meier curve was applied to calculate the survival rates, and the differences in survival of serval group patients were subsequently compared using the log-rank test. A multivariate Cox proportional hazards with “Enter” method was developed to identify the independent risk factors by including the parameters whose *P* value was less than 0.05 in the univariate analysis. The Hazard ratios (HRs) and 95% confidence intervals (CIs) were finally generated. The statistical tests performed above were two-sided; a *P* value less than 0.05 was considered significant.

## Results

### Patient characteristics

The detailed clinicopathologic information of the total patients is shown in Table 1. The median age of all patients was 56 years (range, 19-78 years), and 60.7% of the patients were male. The median tumor size was 4.0 cm (1.0-13.0 cm). The median number of retrieved lymph nodes was 16 (range 2–68), and the median number of metastatic lymph nodes was 2 (range 1–23). Of all the patients, 87 (21.6%) had positive LVI alone, 54 (13.4%) had positive PNI alone, 54 (13.4%) had both positive LVI and PNI, and 207 (51.5%) had both negative LVI and PNI. Accordingly, 141 (35.1%) patients belonged to the LVI-positive group, and 108 (26.9%) patients belonged to the PNI-positive group. Regarding adjuvant chemotherapy, the median cycle of the XELOX regimen was 6 (range, 1-8).

**Table 1:** Clinical and pathological information of total patients in current study

Characteristic	No. (%)
**Age (years)**	
≤ 60	263 (65.4)
> 60	139 (34.6)
**Sex**	
Female	158 (39.3)
Male	244 (60.7)
**Tumor site**	
Cecum	19 (4.7)
Ascending colon	71 (17.7)
Hepatic flexure	38 (9.5)
Transverse colon	42 (10.4)
Splenic flexure	7 (1.7)
Descending colon	32 (8.0)
Sigmoid colon	193 (48.0)
**Tumor size (cm)**	
≤ 4.0	211 (52.5)
> 4.0	191 (47.5)
**T stage**	
1	2 (0.5)
2	15 (3.7)
3	229 (57.0)
4	156 (38.8)
**N stage**	
1	295 (73.4)
2	107 (26.6)
**TNM stage**	
IIIA	11 (2.7)
IIIB	189 (47.0)
IIIC	202 (50.2)
**Tumor differentiation**	
Well/moderate	255 (63.4)
Poor	147 (36.6)
**LVI**	
Positive	141 (35.1)
Negative	261 (64.9)
**PNI**	
Positive	108 (26.9)
Negative	294 (73.1)
**Preoperative CEA (ng/ml)**	
≤ 5	225 (56.0)
> 5	177 (44.0)
**Preoperative CA19-9 (U/ml)**	
≤ 35	323 (80.3)
> 35	79 (19.7)
**Adjuvant chemotherapy cycle**	
< 6	96 (23.9)
6-8	306 (76.1)

*Abbreviations*: *CEA* carcinoembryonic antigen, *CA19-9* cancer antigen 19-9, *LVI* lymphovascular invasion, *PNI* perineural invasion, *TNM stage* clinical tumor-node-metastasis stage

### The relationship of LVI and PNI with clinicopathological features

As shown in Table 2, the presence of both LVI and PNI was positively related to N2 stage (*P* = 0.025; *P* = 0.036) and poorer tumor differentiation (*P* = 0.002; *P* = 0.014). Moreover, LVI was associated with T4 stage (*P* < 0.001). The large tumor size was more frequently found in the patients with PNI-negative tumors (*P* = 0.006). The differences in other parameters between the groups did not show statistical significance.

**Table 2:** Relationships between LVI as well as PNI and patient characteristics

Characteristic	LVI			PNI		
	Positive, ***n*** = 141 (%)	Negative, ***n*** = 261 (%)	***P*** value	Positive, ***n*** = 108 (%)	Negative, ***n*** = 294 (%)	*P* value
**Age (years)**						
≤ **60**	98 (69.5)	165 (63.2)	0.206	70 (64.8)	193 (65.6)	0.877
> **60**	43 (30.5)	96 (36.8)		38 (35.2)	101 (34.4)	
**Sex**						
Female	57 (40.4)	101 (38.7)	0.735	34 (31.5)	124 (42.2)	0.052
Male	84 (59.6)	160 (61.3)		74 (68.5)	170 (57.8)	
**Tumor site**						
Right-sided colon	66 (46.8)	104 (39.8)	0.178	47 (43.5)	123 (41.8)	0.762
Left-sided colon	75 (53.2)	157 (60.2)		61 (56.5)	171 (58.2)	
**Tumor size (cm)**						
≤ 4.0	82 (58.2)	129 (49.4)	0.094	69 (63.9)	142 (48.3)	0.006
> 4.0	59 (41.8)	132 (50.6)		39 (36.1)	152 (51.7)	
**T stage**						
1-3	67 (47.5)	179 (68.6)	< 0.001	61 (56.5)	185 (62.9)	0.240
4	74 (52.5)	82 (31.4)		47 (43.5)	109 (37.1)	
**N stage**						
1	94 (66.7)	201 (77.0)	0.025	71 (65.7)	224 (76.2)	0.036
2	47 (33.3)	60 (23.0)		37 (34.3)	70 (23.8)	
**Tumor differentiation**						
Well/moderate	75 (53.2)	180 (69.0)	0.002	58 (53.7)	197 (67.0)	0.014
Poor	66 (46.8)	81 (31.0)		50 (46.3)	97 (33.0)	
**Preoperative CEA (ng/ml)**						
≤ 5	75 (53.2)	150 (57.5)	0.409	59 (54.6)	166 (56.5)	0.743
> 5	66 (46.8)	111 (42.5)		49 (45.4)	128 (43.5)	
**Preoperative CA19-9 (U/ml)**						
≤ 35	113 (80.1)	210 (80.5)	0.939	87 (80.6)	236 (80.3)	0.949
> 35	28 (19.9)	51 (19.5)		21 (19.4)	58 (19.7)	
**Adjuvant chemotherapy cycles**						
< 6	35 (24.8)	61 (23.4)	0.745	21 (19.4)	75 (25.5)	0.206
6-8	106 (75.2)	200 (76.6)		87 (80.6)	219 (74.5)	

*Abbreviations*: *CEA* carcinoembryonic antigen, *CA19-9* cancer antigen 19-9, *LVI* lymphovascular invasion, *PNI* perineural invasion

### Prognostic value of LVI and PNI for DFS and OS

**Fig. 2:**
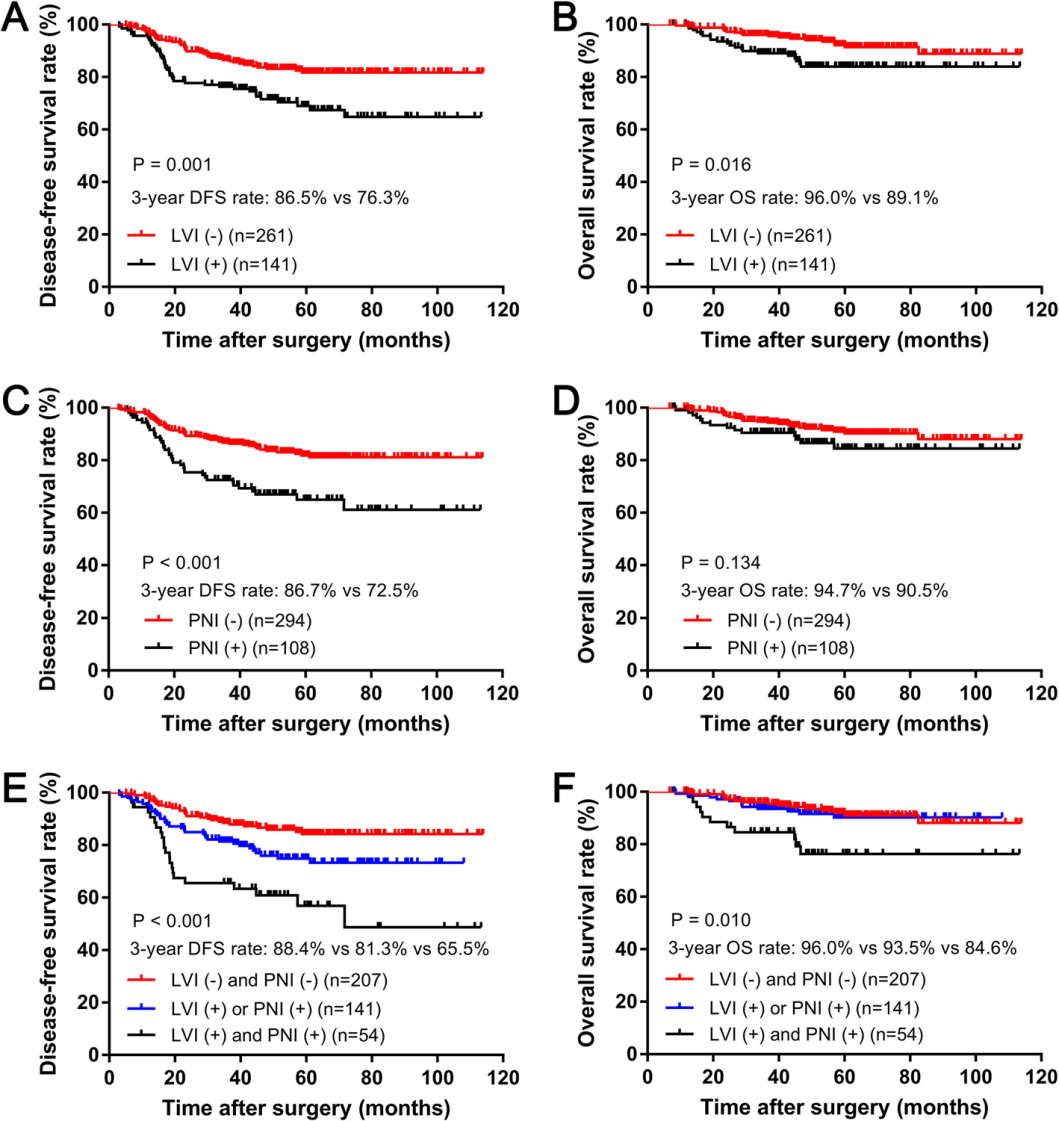
Kaplan-Meier survival analysis of DFS and OS in LVI (+/−) group or/and PNI (+/−) group, respectively

After a median postoperative follow-up duration of 56 months (range, 7–114 months), the 3-year DFS and OS rates were 82.9 and 93.6% in the total enrolled patients in this study. Kaplan–Meier analysis indicated that LVI-positive group showed the significantly worse 3-year DFS and OS rates compared with those in the LVI-negative group (DFS: 76.3% vs. 86.5%, *P* = 0.001, Fig. 2A; OS: 89.1% vs. 96.0%, *P* = 0.016, Fig. 2B). The PNI-positive group presented a significantly lower 3-year DFS rate by comparison to those with the PNI-negative group (72.5% vs. 86.7%, *P* < 0.001, Fig. 2C), while the 3-year OS rate was comparable between the two groups (90.5% vs. 94.7%, *P* = 0.134, Fig. 2D). Patients with the concurrent presence of LVI and PNI had the worst 3-year DFS (65.5% vs. 81.3% vs. 88.4%, *P* < 0.001; Fig. 2E) and OS (84.6% vs. 93.5% vs. 96.0%, *P* = 0.010; Fig. 2F) rates among those with either the presence of LVI and PNI and the absence of LVI and PNI.

### Identification of the independent prognostic factors

The results of univariate and multivariate analyses are summarized in Table 3. The univariate analysis result revealed that male sex, N2 stage, poorly differentiated tumor histology, LVI, PNI, preoperative CEA > 5 ng/ml, and preoperative CA19-9 > 35 U/ml were associated with unfavorable DFS. In contrast, right-sided colon cancer, T4 stage, N2 stage, LVI, preoperative CA19-9 > 35 U/ml, and adjuvant chemotherapy less than six cycles were associated with unfavorable OS. In addition, multivariate result analysis showed that male sex (HR, 1.906; 95% CI, 1.180-3.077; *P* = 0.008), LVI (HR, 1.828; 95% CI, 1.182-2.825; *P* = 0.007), PNI (HR, 1.921; 95% CI, 1.238-2.981; *P* = 0.004), and preoperative CEA > 5 ng/ml (HR, 1.756; 95% CI, 1.142-2.701; *P* = 0.010) were the independent predictive factors for unfavorable DFS, while right-sided colon cancer (HR, 0.427; 95% CI, 0.223-0.815; *P* = 0.010), T4 stage (HR, 2.777; 95% CI, 1.435-5.374; *P* = 0.002) and preoperative CA19-9 > 35 U/ml (HR, 2.472; 95% CI, 1.279-4.780; *P* = 0.007) were the independent predictive factors for unfavorable OS.

**Table 3:** Univariate and multivariate analyses for identifying prognostic factors

	DFS				OS			
	Univariate analysis		Multivariate analysis		Univariate analysis		Multivariate analysis	
	HR (95% CI)	***P*** value	HR (95% CI)	***P*** value	HR (95% CI)	***P*** value	HR (95% CI)	***P*** value
**Age (years)**								
> 60 vs. ≤ 60	1.435 (0.931-2.211)	0.102			1.592 (0.845-2.998)	0.150		
**Sex**								
Male vs. Female	1.808 (1.127-2.900)	0.014	1.906 (1.180-3.077)	0.008	1.567 (0.794-3.095)	0.196		
**Tumor site**								
Left-sided colon vs. Right-sided colon	0.944 (0.614-1.451)	0.792			0.415 (0.218-0.792)	0.008	0.427 (0.223-0.815)	0.010
**Tumor size (cm)**								
> 4.0 vs. ≤ 4.0	0.797 (0.518-1.225)	0.301			0.783 (0.414-1.483)	0.453		
**T stage**								
4 vs. 1-3	1.218 (0.793-1.872)	0.367			2.578 (1.334-4.985)	0.005	2.777 (1.435-5.374)	0.002
**N stage**								
2 vs. 1	1.637 (1.052-2.547)	0.029		0.085	2.316 (1.234-4.349)	0.009		0.060
**Tumor differentiation**								
Poor vs. Well/moderate	1.576 (1.025-2.424)	0.038		0.241	1.330 (0.695-2.546)	0.388		
**LVI**								
Positive vs. Negative	1.985 (1.297-3.037)	0.002	1.828 (1.182-2.825)	0.007	2.122 (1.132-3.979)	0.019		0.109
**PNI**								
Positive vs. Negative	2.250 (1.463-3.461)	< 0.001	1.921 (1.238-2.981)	0.004	1.641 (0.853-3.159)	0.138		
**Preoperative CEA (ng/ml)**								
**> 5 vs.** ≤ **5**	1.690 (1.102-2.592)	0.016	1.756 (1.142-2.701)	0.010	1.217 (0.650-2.281)	0.539		
**Preoperative CA19-9 (U/ml)**								
> 35 vs. ≤ 35	1.690 (1.047-2.727)	0.032		0.115	2.447 (1.271-4.711)	0.007	2.472 (1.279-4.780)	0.007
**Adjuvant chemotherapy cycles**								
6-8 vs. < 6	0.693 (0.432-1.113)	0.129			0.461 (0.239-0.888)	0.021		0.054

*Abbreviations*: *HR* hazard ratio, *CI* confidence interval, *LVI* lymphovascular invasion, *PNI* perineural invasion, *CEA* carcinoembryonic antigen, *CA19-9* cancer antigen 19-9

### Prognostic analysis of the different adjuvant chemotherapy duration with respect to the statuses of LVI and PNI

**Fig. 3:**
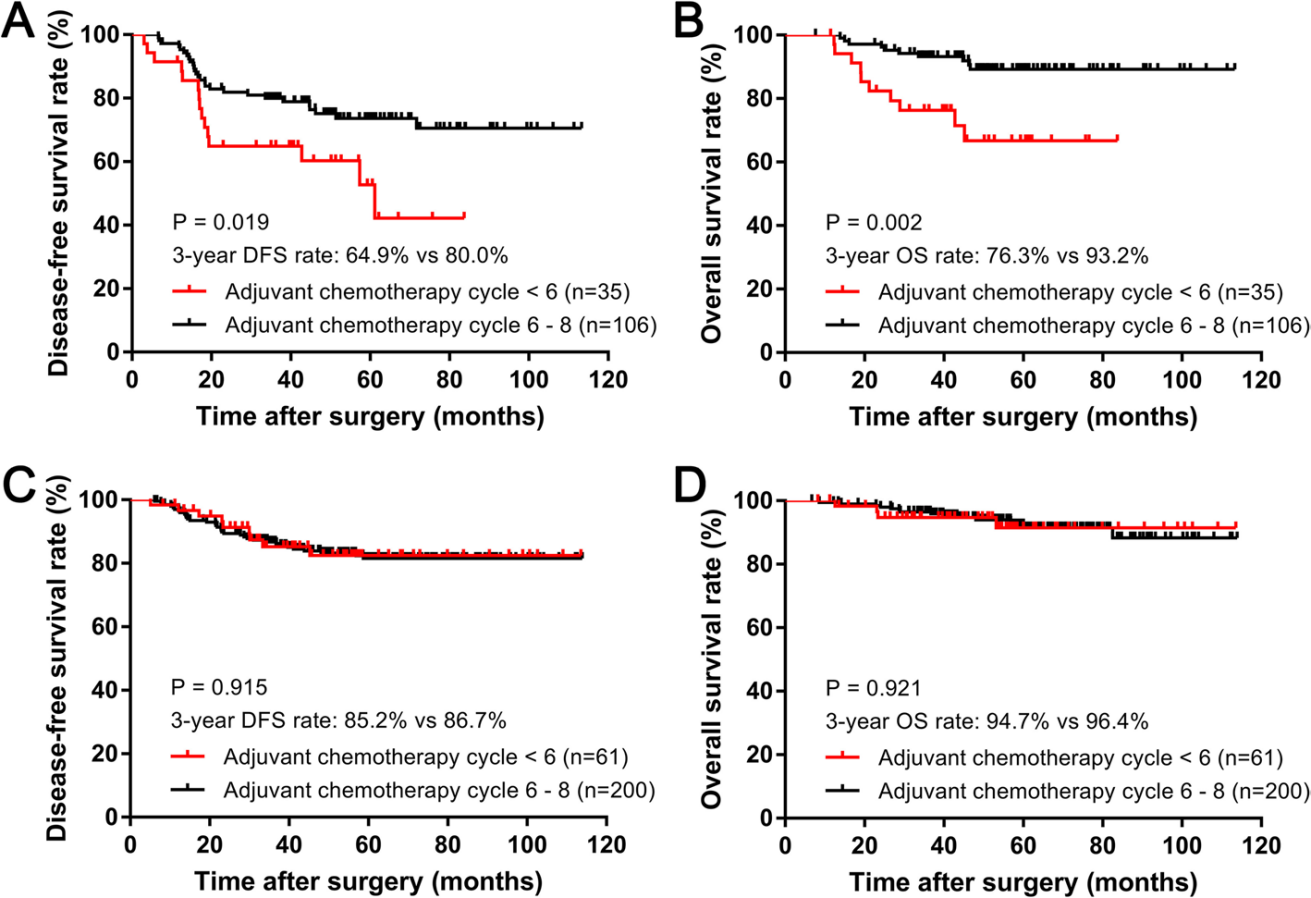
Kaplan-Meier survival analysis of 3-year DFS (**A**) and OS (**B**) rates between treatment circle < 6 and circle 6-8 in LVI-positive group; 3-year DFS (**C**) and OS (**D**) rates between treatment circle < 6 and circle 6-8 in LVI-negative group

Among the LVI-positive group, the patients completing 6–8 cycles of adjuvant chemotherapy presented significantly better 3-year DFS and OS rates than those who completed less than six cycles (DFS: 80.0% vs. 64.9%, *P* = 0.019, Fig. 3A; OS: 93.2% vs. 76.3%, *P* = 0.002, Fig. 3B). For the LVI-negative group, the 3-year DFS or OS rate was comparable between patients who completed 6–8 cycles of adjuvant chemotherapy and those who completed less than six cycles (DFS: 86.7% vs. 85.2, *P* = 0.915; OS: 96.4% vs. 94.7%, *P* = 0.921).

**Fig. 4:**
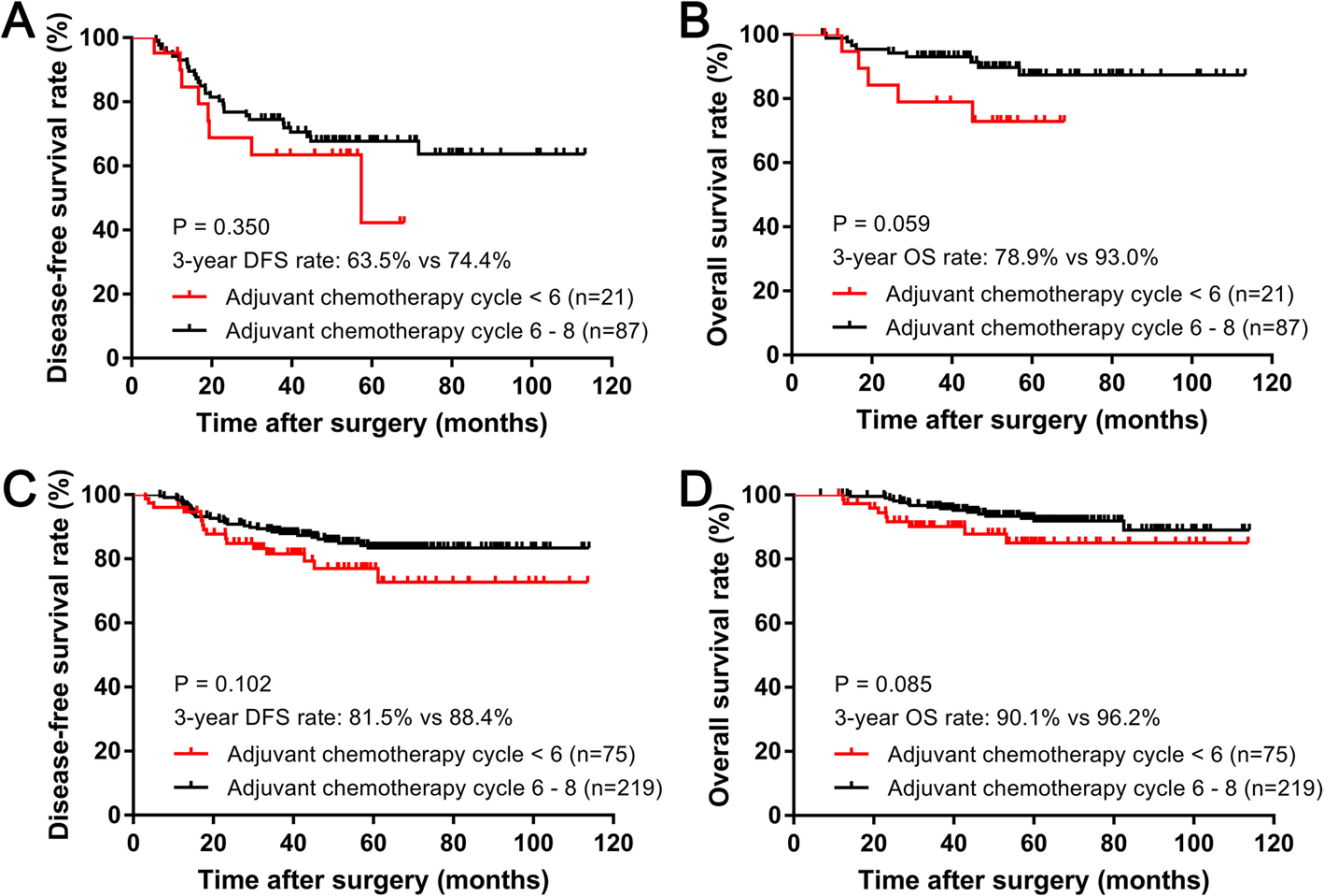
Kaplan-Meier survival analysis of 3-year DFS and OS rates between treatment circle < 6 and circle 6-8 in the PNI-positive/negative group, respectively

However, despite the PNI status, no significant difference was observed in the 3-year DFS or OS rate whether the patients completed 6–8 cycles of adjuvant chemotherapy or less than six cycles (Fig. 4).

## Discussion

It is well known that tumor invasion and the number of metastatic lymph nodes are two vital pathological parameters that can be applied to identify the recurrence risk in stage III colon cancer [23]. In addition to TNM staging, the companion stage diagnosis characterized by other specific pathologic parameters warrants refining stage III colon cancer risk classifications. We assessed LVI and PNI in tumor specimens in the current study and further demonstrated their prognostic value for stage III colon cancer. As a result, two significant findings from this study were noted: (1) LVI and PNI are two important pathological factors for predicting 3-year DFS in patients with stage III colorectal cancer; and (2) LVI is a better therapeutic indicator than PNI, in that LVI can also serve as an influential predictive factor for the efficacy of sufficient duration of adjuvant chemotherapy.

Our data showed that the incidences of LVI and PNI among the 402 patients were 35.1 and 26.9%, respectively, which were higher than those described in previous reports [24, 25]. The frequency of LVI and PNI is stage-dependent and indicates the likelihood of lymph node involvement. Previous studies have noted that PNI involvement was present in only 9.5% of stage I and II CRC tumors compared with 26.3% of stage III tumors and 36.6% of stage IV CRC tumors. Similarly, the presence of LVI also increased from 5.5% in stage I tumors to 24.4% in stage IV tumors [26, 27]. In addition, another study revealed that the presence of LVI or PNI was the independent risk factor for lymph node metastasis in colorectal cancer [28]. Similarly, our findings revealed that the presence of LVI and PNI had a positive association with advanced N stage, which indicates an adverse clinical course in stage III colon cancer. Although the causality of LVI status and lynph node metastasis was not clear enough. Histologically, LVI is an early and necessary step for lymphatic metastasis. However, despite the presence of lymph node metastasis, there are more than a few cases with negative LVI, and in the present study, 261 of 402 cases (64.9%) were positive for lymph node metastasis. We consider LVI status develop via a part of mechanism of lynph node positibility. Thus, these findings suggest that the LVI and PNI were indicators for more extensive surgical tumor resection.

To our knowledge, the unfavorable prognostic impact of LVI and PNI in lymph node-negative colorectal cancer patients has been well identified [19, 20, 25]. Therefore, current practice guidelines suggest that adjuvant chemotherapy is recommended for stage II colon cancer patients with poor prognostic characteristics, including PNI and LVI. Recently, Zhong JW et al. revealed that LVI was an indicator of more aggressive biological behavior and an unfavorable prognosis in patients with stage III colorectal cancer [22]. However, there were two limitations to the study by Zhong JW et al. that could not confirm the prognostic value of LVI for stage III colon cancer. The study included not only colon cancer patients but also rectal cancer patients. Previous studies have revealed different recurrence patterns between LVI-positive colon cancer and LVI-positive rectal cancer, indicating that LVI’s prognostic properties existed discrepancies in colon and rectal cancer [29].

Moreover, information on postoperative treatment is unavailable, which underestimates the prognostic value of LVI. Unlike the study by Zhong JW et al., our present study only focused on stage III colon cancer and performed unified curative resection followed by adjuvant chemotherapy with the XELOX regimen. Subsequently, we confirmed the presence of PNI, which indicated poor 3-year DFS in these patients. Fujita S et al. were the first to report that the PNI-positive group presented a significantly worse DFS rate than the PNI-negative group in stage III colon cancer, despite adjuvant chemotherapy administration [21]. Our study revealed that the presence of PNI indicated worse 3-year DFS than the absence of PNI and was also an independent risk factor. While the PNI status seemed didn’t influence the 3-year OS in these patients. From this perspective, the PNI status is inferior to LVI status in prognostic value. Moreover, we also identified a subgroup of patients with the presence of both LVI and PNI who showed the worst 3-year DFS and OS rates, revealing the prognostic superposition of the two pathological parameters in colon cancer.

Here, LVI and PNI, serving as unfavorable prognostic factors in colon cancer, might mainly attribute to the aggressive tumor type. Our findings indicate that LVI and PNI are significantly related to poor pathological differentiation and an advanced tumor stage. Similarly, previous data have shown that both LVI and PNI were closely related to the aggressive tumor features characterized by poor pathological differentiation and an advanced tumor stage [30, 31]. Jiang HH et al. revealed that the presence of LVI was correlated with genomic alterations activating aggressive tumor behavior, such as angiogenesis, epithelial-mesenchymal transition, and matrix remodeling [27]. Kim JC et al. reported that the existence of PNI was closely associated with the expression of gelsolin which promoted tumor cell proliferation and migration by degrading the extracellular matrix and subsequently contributes to the systemic recurrence of colorectal cancer [32]. Understanding the potential mechanisms underlying this association might help improve future therapeutic strategies to inhibit the metastatic spread of cancer with LVI and PNI.

Interestingly, LVI exhibited prognostic value and predictive power in response to adjuvant chemotherapy, while PNI had only prognostic value in 3-year DFS for stage III colon cancer. A recent study found that PNI can serve as a prognostic indicator but not a predictive indicator for adjuvant chemotherapy efficacy for colon cancer [33]. Accordingly, the presence of LVI is a more crucial pathological risk factor than PNI. Our findings revealed that treatment benefits from the total planned duration of adjuvant chemotherapy were only observed in patients in the LVI-positive group but not in the LVI-negative group. Therefore, a novel strategy based on the presence of LVI can be developed for individual management of adjuvant chemotherapy for stage III colon cancer. Once patients are found to have LVI, sufficient duration of adjuvant chemotherapy should be recommended, whereas for patients without LVI, an entire course of adjuvant chemotherapy is better to avoid chemotherapy-related toxicity.

Several limitations to the current study were acknowledged. First, this retrospective study was performed with an uncontrolled methodology by including a limited number of patients in a single cohort. Although our study initially indicated the potential prognostic value of LVI and PNI, the findings must be validated in a prospective, multicenter clinical trial with a large population in the future. Second, the median 56-month follow-up duration had insufficient power to calculate 5-year survival outcomes, which might result in a misestimation of the effect of LVI and PNI on OS. Additionally, tumor molecular markers, such as the microsatellite status, the CpG island methylator phenotype (CIMP) status, driver gene mutations, such as KRAS and BRAF, and tumor immune microenvironment, have been linked to different recurrence risks of stage III colon cancer [34, 35]. The above molecular data were unavailable in the current study. Thus, it is necessary to include molecular prognostic markers for risk stratification in further studies.

## Conclusion

Our study confirms that LVI represents a superior prognostic factor to PNI for stage III colon patients undergoing curative resection followed by adjuvant chemotherapy. PNI status can noly predict the 3-year DFS wihout affecting the 3-year OS. Moreover, our findings also indicate the predictive value of LVI as an efficacy indicator for adjuvant chemotherapy duration.

## Data Availability

The authenticity of this article has been validated by uploading the key raw data onto the Research Data Deposit public platform (http://www.researchdata.org.cn), with the approval number as RDDA2019001198.
